# Contemporary Theory and Research

**Published:** 1893-04-29

**Authors:** 


### Contemporary Theory and Research.

### ASAPROL.

According to Dr Stachler, asaprol is the calcium salt of B naphthol and monosulphonic acid, and occurs in the form of a white powder, very soluble in cold water. At 122 deg. F., it undergoes decomposition. Asaprol is toxic to rabbits in large doses. Smaller doses are well borne. Clinically, Dr. Stachler has tested it in two cases of influenza with high temperature and great pain with good results. In one case all symptoms were relieved in 36 hours and in the other in 48 hours, though both quinine and antipyrin had been given without avail. The drug proved equally efficient in the various forms of rheumatism, especially in acute articular rheumatism. Dr. Stachler has also found it efficacious in other affections, such as gout, asthma, tonsilitis, &c. Gradually increasing doses are recommended. Asaprol is incompatible with alkaline iodides, sulphates, and with most of the alkaline salts.

### THE DANGERS OF BANK-NOTES.

Dr. Acosta and Dr. Grande Rossi have been investigating the bank-notes of Havanah, and have published the following report: "1. In the Spanish bank-notes of Havanah, which have been in circulation some time, a considerable number of microbes may be detected. On two notes—the one for ten, the other for five, 'centavos'—we counted no less than 19,147. 2. On the notes submitted to our examination there existed a septic species which rapidly killed turkeys when duly inoculated. 3. Other pathogenic species (diphtheria, tuberculosis, &c.), might easily be met with on the said notes. 4. These notes form thus a powerful means of contagion, and their use is, above all, dangerous for children, who might carry them to their lips, swallow the microbes, and acquire in this manner mortal diseases." The dangers to which children are exposed in this sucking of bank notes are undoubtedly grave, but how about the danger to the notes?

### PROLONGED INCUBATION OF MUMPS.

M. Merklen has presented to the Société Médicale des Hôpitaux a report on the contagiousness of mumps. After a prolonged observation he fixes the period of incubation at from 15 to 26 days. This long period presents a certain practical interest as explaining the considerable extension and long duration of epidemics of mumps under certain circumstances in barracks, boarding-schools, &c. No actual facts decide at what precise moment the contagion ceases, but all observations tend to show that the contagion survives the malady. The practical conclusion is that it is prudent to maintain the complete isolation of the patients until the recovery is perfect, and to use the same measures of precaution in their disinfection as for other contagious maladies.

### TROPACOCAINE.

This preparation, known also as Benzoyle Pseudotropeine is an alkaloid obtained from the leaves of the small-leaved coca of Java. It has been found by Dr. Hugenschmidt, a Parisian dentist, to be much less toxic than cocaine and equally efficacious. He employs the following solution: Tropacocaine, 10 centigrammes; distilled water, 2 grains 50 centigrammes; ten drops to be injected, 25 milligrammes. Allowing one minute for the injection into the tissues no toxic symptoms are caused, and the extraction of teeth and other dental operations are rendered entirely painless. The hydrochlorate of tropacocaine being a synthetic product, is much less liable to vary in its composition than the natural product. Dr. Hugenschmidt thinks that the greater toxicity of cocaine remarked during the last few years may be accounted for on the supposition that the cocaine formerly in use was obtained from old leaves, while the more modern cocaine is extracted from fresh leaves. It is claimed for tropacocaine that while it is even more efficient as a local anæsthetic than cocaine, it is far less toxic, and moreover keeps well in solution for months, while cocaine tends to decompose and lose its properties at the end of a few days.

